# Mandibular facial talon cusp: Case report

**DOI:** 10.1186/1472-6831-5-9

**Published:** 2005-12-08

**Authors:** Folakemi A Oredugba

**Affiliations:** 1 Department of Child Dental Health, College of Medicine University of Lagos PMB 12003 Idi-Araba Lagos, Nigeria

## Abstract

**Background:**

Talon cusp is a supernumerary structure projecting from the dento-enamel junction to a variable distance towards the incisal edge of an anterior tooth. Studies have shown that it consists of enamel, dentine and a variable amount of pulp tissue. Hyperactivity of the enamel organ during morphodifferentiation has been attributed to its formation. Most previous reports have been made concerning the occurrence of this structure on primary and permanent teeth and mostly on the palatal aspect. Only few have been reported on the facial aspect of the teeth. When it occurs, the effects are mainly aesthetic and functional and so early detection and treatment is essential in its management to avoid complications.

**Case presentation:**

An unusual case of talon cusp on the facial aspect of a mandibular central incisor is reported. Its presence resulted in attrition of the opposing tooth. Reduction of the cusp and topical application of fluoride gel was initiated.

**Conclusion:**

The management and treatment outcome of talon cusp depends on the size, presenting complications and patient cooperation.

## Background

This unusual dental anomaly showing an accessory cusp-like structure projecting from the cingulum to the cutting edge was first described by Mitchell in 1892 [1]. It was thereafter named a Talon cusp by Mellor and Ripa [2] due to its resemblance to an eagle's talon. Since then, this odontogenic anomaly has been given several descriptions, such as, prominent accessory cusp-like structure [3], exaggerated cingula [4] additional cusp [5], cusp-like hyperplasia [6], accessory cusp [7] and supernumerary cusp [8]. It has been defined as a supernumerary accessory talon-shaped cusp projecting from the lingual or facial surface of the crown of a tooth and extending for at least half the distance from the cemento-enamel junction to the incisal edge [7]. There is a wide variation in the size and shape of this anomaly. Due to this variation, and in order to have a diagnostic criteria, it has been classified into 3 types by Hattab et al [9]:

Type1: Talon – refers to a morphologically well-delineated additional cusp that prominently projects from the palatal (or facial) surface of a primary or permanent anterior tooth and extends at least half the distance from the cemento-enamel junction to the incisal edge.

Type 2: Semi talon – refers to an additional cusp of a millimeter or more extending less than half the distance from the cemento-enamel junction to the incisal edge. It may blend with the palatal surface or stand away from the rest of the crown.

Type 3: Trace talon – an enlarged or prominent cingula and their variations, i.e. conical, bifid or tubercle-like.

Radiographically, it may appear typically as a v-shaped radiopaque structure, as in true talon or semi- talon, or be tubercle-like, as in trace talon, originating from the cervical third of the root. The radiopaque v-shaped structure is superimposed over the normal image of the crown of the tooth. The point of the 'V' is inverted in mandibular cases. This appearance varies with the shape and size of the cusp, and the angle at which the radiograph is taken.

It is composed of enamel, dentine and a varying amount of pulp tissue [10,11]. The extent of pulp extension into the cusp is however difficult to determine because of its superimposition over the main pulp chamber [12]. While some indicated that talon cusps contain pulp tissue [2,10,13], some found no evidence of pulp extension into the cusp [14,15]. However, it has been suggested that large talon cusps, especially those that stand away from the tooth crown are more likely to contain pulp tissue [9,12]. A review of the literature showed that over the last two decades, increasing reports have been made of the occurrence of the condition. The reported prevalence outside Africa is between 0.06% in Mexicans [16] and 7.7% in a northern Indian population [17]. It has also been found to be relatively common in the Chinese [5,6] and Arab [9], and predominantly in the male population [18]. These wide variations in prevalence could be due to individual differences in definitions of observation, from enlarged cingula to semi- or true talons [17]. If data is taken from those who reported for treatment only, a high prevalence might be observed. Patients may seek treatment when there is a problem, usually with large cusps. No prevalence data has been found in the literature for Africans.

### Aetiology

The exact aetiology is not known, but it is suggested to be a combination of genetic and environmental factors [9,19,20]. It is thought to arise during the morphodifferentiation stage of tooth development, as a result of outfolding of the enamel organ or hyperproductivity of the dental lamina [9,21]. It is suggested that disturbances during morphodifferentiation such as altered endocrine function might affect the shape and size of the tooth without impairing the function of ameloblasts and odontoblasts [22]. There is also a suggestion of a strong genetic influence in its formation as evidenced by its occurrence in close family members [18,20,23-25]. Talon cusp may occur in isolation or with other dental anomalies such as mesiodens [3], odontome, unerupted or impacted teeth [13,26], peg-shaped maxillary incisor [26], dens invaginatus [26-28], cleft lip and distorted nasal alae [29], bilateral gemination [18,30], fusion [31,32], supernumerary teeth and enamel clefts [33,34]. It has also been associated with some systemic conditions such as Mohr syndrome (oro-facial-digital II) [35], Sturge-Weber syndrome (encephalo-trigeminal angiomatosis) [6], Rubinstein-Taybi syndrome [36], incontinentia pigmenti achromians [37] and Ellis-van Creveld syndrome [38].

### Presentation

It is more common in the permanent dentition (75%) than in the primary dentition, while 92% affect the maxillary teeth [8,9]. The maxillary lateral incisor is the most frequently affected in the permanent dentition while the maxillary central incisor is the most affected in the primary dentition [8]. Most times it occurs unilaterally but bilateral cases, including multiple talon cusps have also been reported [3,6,9,24,25,33,39]. In a particular case, talon cusps have occurred on both maxillary and mandibular teeth in the same patient [11]. Rarely, two talon cusps may occur on a single tooth. Abbot reported a labial and a palatal talon on a maxillary right central incisor [40], while another report from Nigeria presented two palatal talons on a maxillary left central incisor [39].

### Complications and management

The complications of talon cusp are diagnostic, functional, aesthetic and pathological [3,41]. A large talon cusp is unaesthetic and presents clinical problems. It may present diagnostic problems if it is unerupted and resembles a compound odontome or a supernumerary tooth and so leads to unnecessary surgical procedure. Functional complications include occlusal interference, trauma to the lip and tongue, speech problems and displacement of teeth. The deep grooves which join the cusp to the tooth may also act as stagnation areas for plaque and debris, become carious and cause subsequent periapical pathology [2,3,41].

Management will depend on individual presentation and complications. Small talon cusps are asymptomatic and need no treatment [24,33]. Where there are deep developmental grooves, simple prophylactic measures such as fissure sealing and composite resin restoration can be carried out [2,13,42-44]. An essential step, especially in case of occlusal interference, is to reduce the bulk of the cusp gradually and periodically and application of topical fluoride such as Duraphat ^® ^or Acidulated Phosphate Fluoride (APF) gel to reduce sensitivity and stimulate reparative dentine formation for pulp protection [45], or outright total reduction of the cusp and calcium hydroxide pulpotomy [46]. It may also become necessary sometimes, to fully reduce the cusp, extirpate the pulp and carry out root canal therapy [19]. Orthodontic correction may become necessary when there is tooth displacement or malalignment of affected or opposing teeth [14,47].

This is a report of an unusual case of talon cusp which presented on the facial aspect of a mandibular central incisor.

## Case presentation

**Figure 1:**
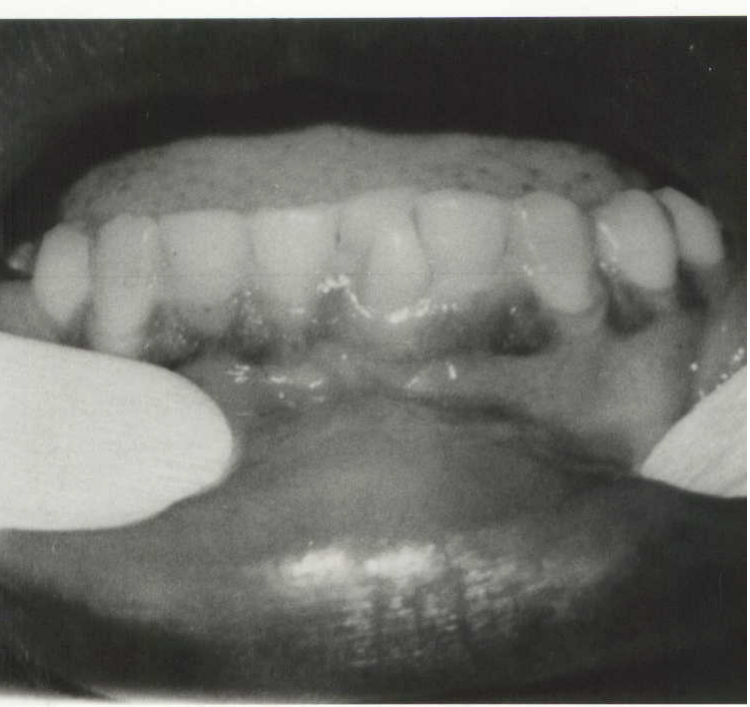
Intra-oral photograph showing the facial talon cusp and lingual displacement of the mandibular left central incisor.

**Figure 2:**
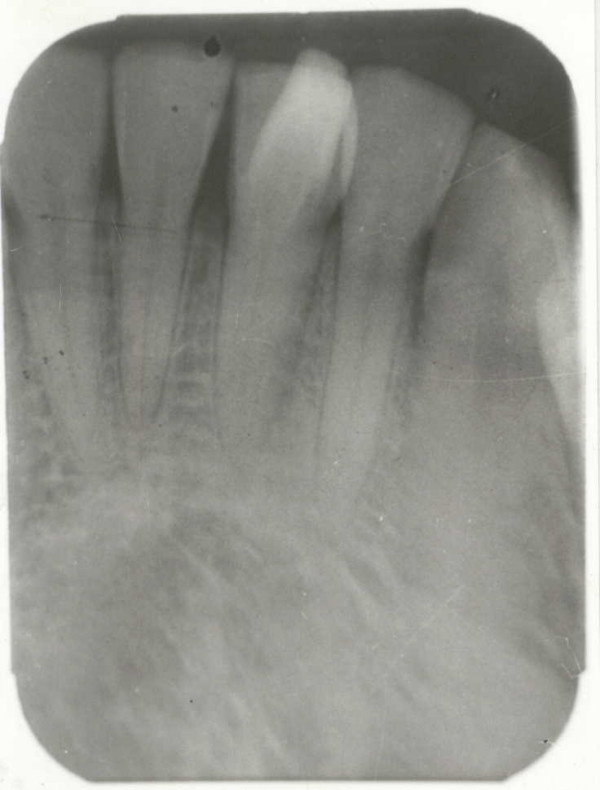
Peri-apical radiograph of the mandibular left central incisor showing the inverted V-shaped talon cusp.

A healthy looking 29 year old Nigerian male presented at the dental outpatient clinic of the Lagos University Teaching Hospital for the purpose of a dental check-up. It was his first visit to the dentist. He did not present any significant medical history. Oral examination showed a fair oral hygiene, no carious lesion, and all the permanent teeth were present. The mandibular left central incisor was displaced lingually with an accessory cusp on the facial aspect which had an attrition facet on the incisal edge. The cusp projected from the cemento – enamel junction and curved towards the incisal edge of the incisor (Figure 1). There was also attrition of the incisal half of the palatal aspect of the maxillary left central incisor. There was a negative family history of such dental anomaly from the patient and there was no associated systemic disorder. A periapical radiograph revealed an inverted V-shaped radiopaque structure on the mandibular left central incisor (Figure 2). The extent of pulp tissue into the cusp could not be determined on the radiograph. A diagnosis of type 1 talon cusp was made. The condition and the planned periodic and gradual reduction of the cusp with topical fluoride application and Composite resin facing was explained to the patient. Orthodontic alignment of the displaced central incisor was also planned. With his consent, after oral prophylaxis, a minimal reduction of the talon cusp was carried out using a diamond bur in a high-speed water-cooled handpiece. Acidulated Phosphate Fluoride (APF) gel was applied to the surface of the reduced cusp and the maxillary left central incisor. The patient however failed to turn up for further treatment. It was assumed that as the patient was not initially concerned with the aesthetic effect of the cusp, the outcome was not important to him.

## Discussion

Reports of mandibular talon cusps are rare in literature. Only ten had been reported, including the present case, with only one on a primary incisor [Table 1]. [11,15,41,48-52]. It is agreed that it is more common in maxillary teeth. Facial talons are also rare: only six cases having been reported before this case [7,37,40,49,52]. It is even more rare in mandibular teeth.

**Table 1:** Reported cases of mandibular talon cusps

Author	Tooth type	Tooth surface
Goel et al, 1976 [15]	Mandibular R 1	Lingual
Mader, 1982 [41]	Mandibular R 1	Lingual
Falomo, 1985 [48]	Mandibular R 2	Lingual
McNamara et al, 1997 [49]	Mandibular R 1	Facial
Hegde and Kumar, 1999 [50]	Mandibular L b Mandibular L 1	Lingual Lingual
Nadkarni et al, 2002 [51]	Mandibular R 1	Lingual
Dash et al, 2004 [11]	Mandibular R 1	Lingual
Llena-Puy and Navarro, 2005 [52]	Mandibular L 2	Facial
Oredugba (Present report)	Mandibular L 1	Facial

There was no associated systemic or local condition in this patient as is the case in most previous reports. Most cases occur in isolation of other conditions [53]. The patient in this report also did not give a history of its occurrence in any member of his family. Of all the cases reported from Nigeria, only two females were affected. This finding supports earlier reports of a higher prevalence of the condition in males. Mays reported a statistically significant bias in favour of males [54].

The present case is a type 1 talon. Although such large cusps which stand away from the tooth had been shown to contain an extension of the pulp, superimposition of the image of the cusp over the main tooth made it difficult to determine the extent of pulp tissue in the anomalous cusp. The constant attrition on the cusp may also mean that there may be reparative dentine which would have taken up part of the pulp space in the cusp.

The presence of a talon cusp is not always an indication for dental treatment unless it is associated with problems such as compromised aesthetics, occlusal interference, tooth displacement, caries, periodontal problems or irritation of the soft tissues during speech or mastication [3,7,42]. Occlusal interference can damage the periodontium, cause infra-occlusion of the opposing tooth and also temporo-mandibular joint pain [25,55]. Severe attrition or fracture of the enamel surface can cause exposure of the dentine-pulp complex and consequently, pulp necrosis [56-58]. In this case, the cusp was prominent and sharply defined and projected from the cervical region to the incisal edge of the tooth. This resulted in occlusal interference, which caused the attrition of the tip of the cusp and the opposing maxillary incisor, and displacement of the mandibular central incisor. The patient was however less concerned due to the painless complications. This explained the lack of compliance with appointment. It is necessary to evaluate and treat talon cusps soon after eruption to prevent these complications.

## Conclusion

The management and treatment outcome of talon cusp depends on the size, presenting complications and patient cooperation.

## Competing interests

The author(s) declare that they have no competing interests.

## Pre-publication history

The pre-publication history for this paper can be accessed here:


